# Improving the confidence level and surgical skills of undergraduate medical students by using pig-perineum simulation-based learning

**DOI:** 10.1186/s12909-025-06771-1

**Published:** 2025-02-14

**Authors:** Sasivimol Srisukho, Chailert Phongnarisorn, Opas Sreshthaputra, Wirawit Piyamongkol, Theera Tongsong

**Affiliations:** 1 Division of Female Pelvic Medicine and Reconstructive Surgery, Department of Obstetrics and Gynecology, Faculty of Medicine, Chiang Mai University, Chiang Mai, 50200 Thailand; 2 Department of Obstetrics and Gynecology, Faculty of Medicine, Chiang Mai University, Chiang Mai, 50200 Thailand

**Keywords:** Pig-perineum, Simulation-based learning, Undergraduate medical students, Confidence level, Surgical skills

## Abstract

**Background:**

In many developing countries facing medical personnel shortage, including Thailand, medical graduates are required to have enough confidence and competence to perform essential procedures by themselves. They should be competent in episiotomy and perineal repair. In the early phase of actual practice of episiotomy on real patients, new clinical students are usually unskilled and less confident. To improve medical students’ competency and confidence in performing basic procedures, we developed a pig-perineum simulation-based learning program. The aim of this study is to evaluate the confidence levels of medical students as well as their perspectives regarding the effectiveness of pig-perineum simulation-based learning.

**Methods:**

Medical students participated in a pig-perineum repair workshop on identifying perineal laceration, knot-tying, and perineal laceration repair, under the instruction of skilled staff members. The workshop was meant to achieve transfer of skills from the simulated setting of our course to the actual practice on patients in the labor ward and the maternity operating room. All the participants were instructed to answer the questions on a questionnaire after the simulation. The confidence, skills, satisfaction, and learning achieved through the simulator were assessed using a 5-point Likert scale, based on the systematically validated questionnaire.

**Results:**

Seven hundred and ninety-two medical students were enrolled in the study. The mean self-efficacy scores before and after the simulation were 3.63 and 4.16 respectively (paired t-test, *p*-value < 0.001). The mean satisfaction score was high (4.22), inspiring them to enhance their surgical skills in knot-tying and perineal laceration repair. The mean self-evaluation score of the participants regarding their perspectives on the closeness to reality of pig-perineum repair when compared to real clinical practice was high (4.09), making them more confident in practice in the labor room.

**Conclusion:**

Pig-perineum simulation-based learning improves confidence level and surgical skills in undergraduate medical students. This approach appears to enhance competency during clinical practice.

**Supplementary Information:**

The online version contains supplementary material available at 10.1186/s12909-025-06771-1.

## Background

Vaginal birth, especially in nulliparous women, is often associated with perineal tear and perineal lacerations. Sometimes, a perineal injury can extend deeper to the anal sphincter and rectal mucosa, known as an obstetric anal sphincter injury (OASI)Waldman [1]. Perineal laceration, either occurring spontaneously or resulting from a surgical incision (episiotomy), is a common obstetric injury, occurring in nearly 90% of women with spontaneous vaginal delivery [2]. Most lacerations heal without long term complications, but some lacerations can lead to prolonged pain, sexual dysfunction and embarrassment [3]. Some lacerations need to be carefully identified and properly repaired at the time of delivery [4].

According to the Medical Council of Thailand, medical students must be taught to perform normal labor, episiotomy and perineorrhaphy or repair of episiotomy wound after normal vaginal delivery. Medical graduates must be capable of effectively performing these procedures by themselves. However, perineal-repair surgical skills are challenging and need special techniques, not gained from general suturing practice. Unfortunately, the acquisition of those skills by medical students is usually hindered by time pressure and a long learning curve. Additionally, a recent survey reported that Thai medical graduates did not achieve the satisfactory confidence levels in performing normal vaginal delivery and episiotomy repair [5].

Furthermore, perineal repair needs complex surgical skills, and the process may cause medical accidents, such as needle-stick injuries, especially in inexperienced trainees. A survey from Sri Lanka reported that 70% of medical students had experienced such events during suturing of episiotomy wounds [6]. Therefore, medical schools need to provide programs that encourage medical students to practice these surgical skills.

Additionally, performing tasks on live patients often cause anxiety and stress for new clinical students because of fear and lack of prior experience. Considering that this procedure involves a long learning curve, which means that much effort is needed and learning is initially difficult, the importance of training stands out. Students who receive simulation training participate more actively in the clinical environment during the course of clerkship. Simulation training is beneficial because it provides a safe environment for learning and practising obstetric skills with minimal risk, increases competency with maneuvers, and translates this competence into increased clinical participation and confidence without any harm to patients [7].

Various teaching models in perineal repair for medical and midwifery students have been proposed, for example using a sponge model, a perineum-padsicle model, a porcine tongue simulator, and a beef tongue simulation model. These teaching modules are aimed at helping students, residents and other practitioners enhance their skills, so that they are able to effectively perform perineal repair, especially in cases of third- or fourth-degree lacerations (OASI).

Previous studies on a porcine tongue simulator [8] and a detailed beef tongue simulation model [9–11] showed that repair workshop for residents could help improve their background knowledge and skills in repairing obstetric anal sphincter injury. In addition, a sponge model improved the repair skills of students regarding second-degree perineal laceration [12], as measured by the Objective Structured Assessment of Technical Skills (OSATS), and fourth-degree perineal laceration [13]. We hypothesize that using real pig perineum for perineal repair practice better represents the human perineum compared to other parts of the animal body or artificial tissue. Accordingly, this preliminary study may provide a new simulation model that is theoretically superior to previously used models.

Since it is essential for medical students to learn basic surgical techniques, as mentioned above, we developed a hands-on perineal repair workshop, using real pig perineum as a model, to teach medical students before they perform the procedure in real clinical practice. The objective of this study is to report the confidence levels of the medical students as well as their perspectives on the closeness to reality of pig-perineum as a simulator.

## Methods

A retrospective study of pig-perineum simulation-based learning (SBL) for medical students was conducted at the Department of Obstetrics and Gynecology, Faculty of Medicine, Chiang Mai University from June 2016 to June 2023, with ethical approval by the Institutional Review Board, Faculty of Medicine, Chiang Mai University (Research ID: OBG-2567-0283), in accordance with the ethical standards of the 1964 Declaration of Helsinki.

### Hands-on pig-perineum simulation-based learning workshop (Fig. 1)

This workshop was designed by the educational team of our department, and its implementation commenced in the 2015 academic year in the obstetric clerkship at the Faculty of Medicine, Chiang Mai University. Medical students participated in a pig-perineum repair workshop on identifying perineal laceration, knot-tying, and perineal laceration repair. The pig perineum used in the workshop was obtained from discarded pork products processed by Betagro Northern Agro Industry Company Limited. The workshop did not involve the use of live animals, nor were any animals commercially purchased or client-owned. These discarded materials, which would have otherwise been destroyed, were repurposed as educational tools.

**Fig. 1:**
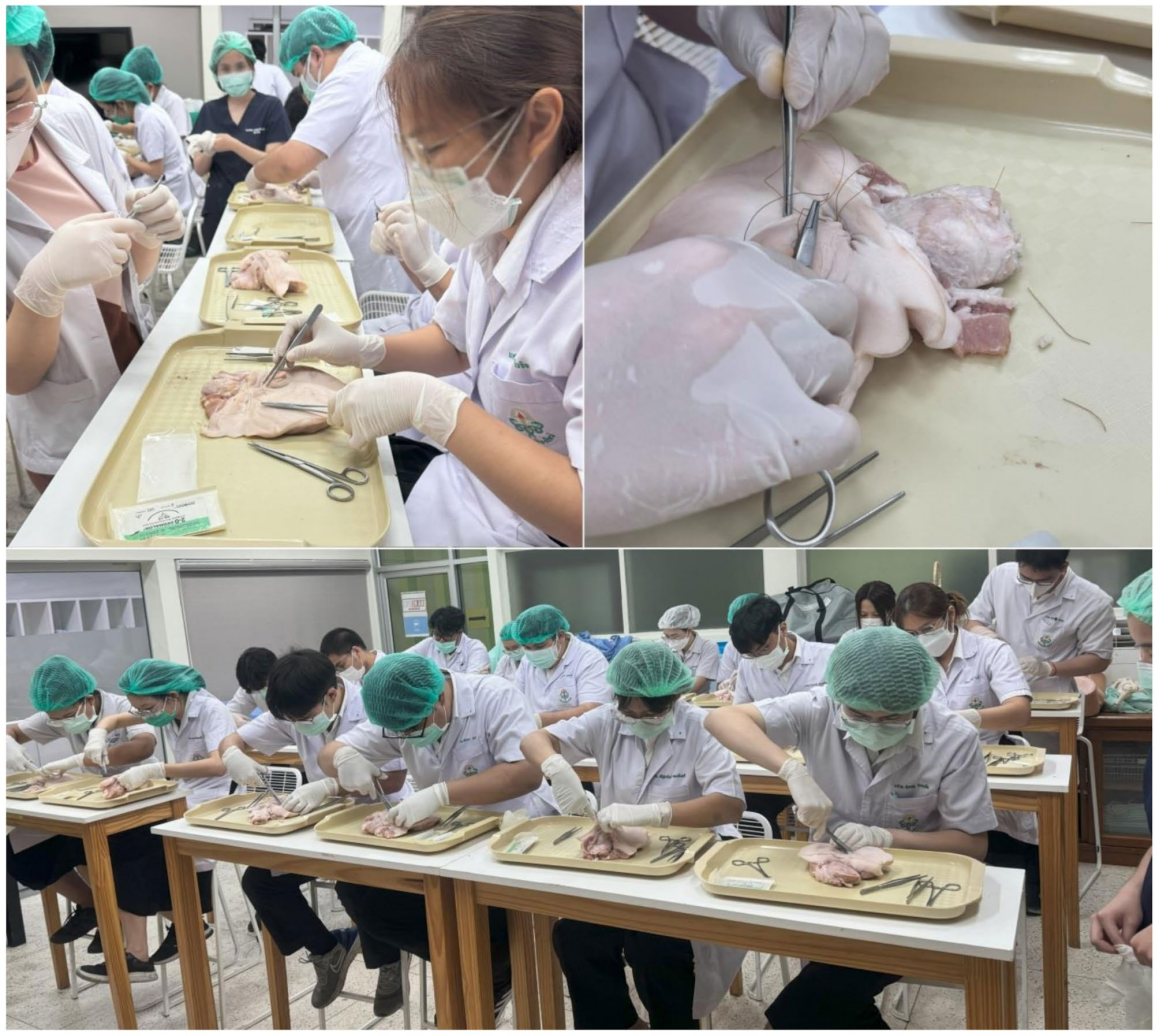
Hands-on pig-perineum simulation-based learning workshop

**Fig. 2:**
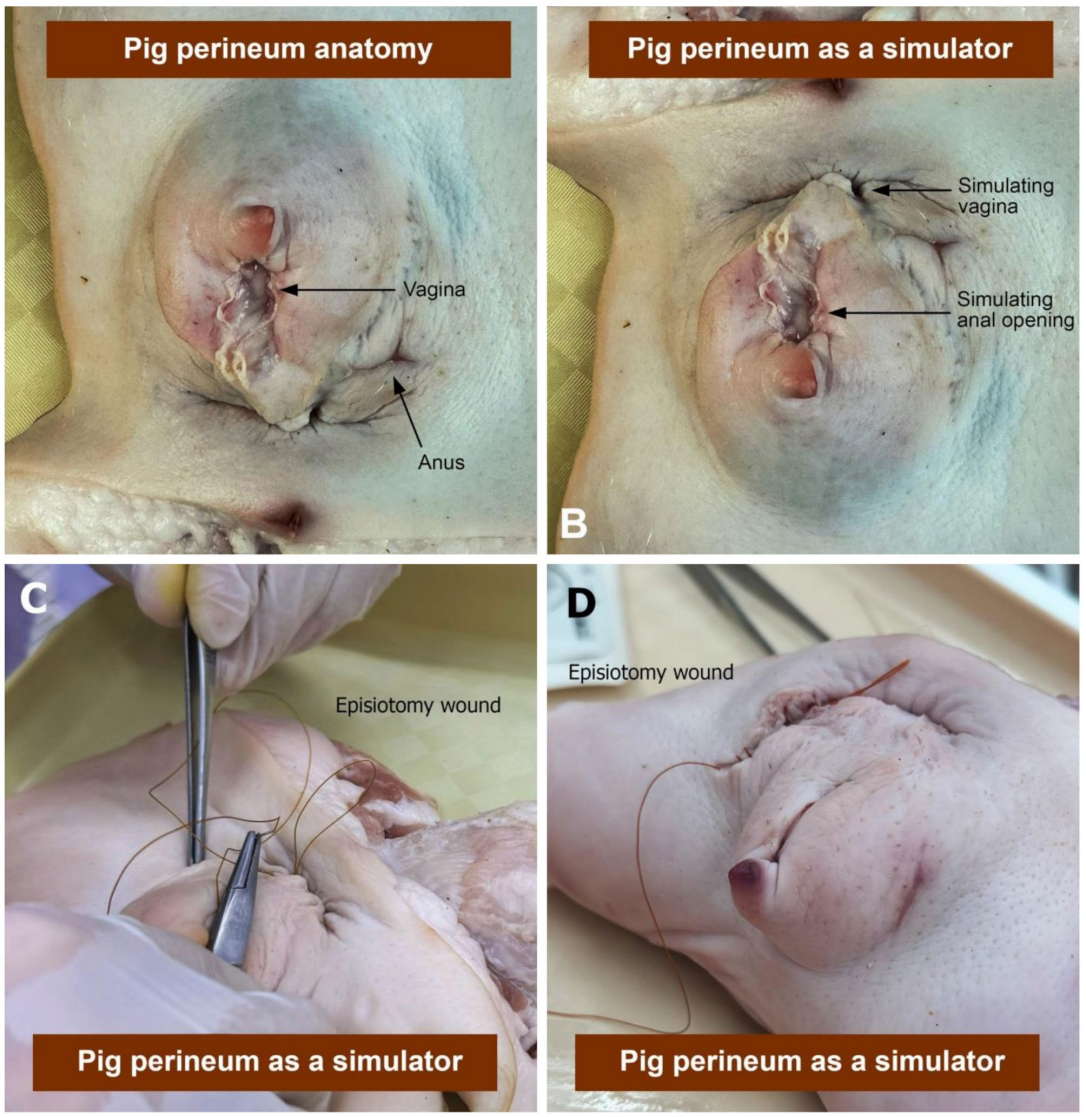
Female pig perineum tissue blocks. (**A**: Female pig perineum anatomy; **B**, **C** and **D**: Diagram of female pig perineum tissue as a simulator)

A half-day training program was conducted for all 5th- and 6th-year medical students who underwent obstetric clerkship rotation. The training program consisted of both lectures and practical training. Students had individual workstations. The first part of the training involved lectures on the following aspects: the anatomy of the perineum, episiotomy, accurate diagnosis of perineal tears, classification and identification of internal and external anal sphincter muscles, and methods of repairing second-degree perineal tears. We used real female-pig perineum tissue blocks as simulators, as shown in Fig. 2. Regarding anatomy, the porcine vaginal diameter is smaller than that of humans. We oriented the (pig) vagina to be an anal opening (simulator) and presumed the anal sphincter to be a perineal muscle (simulator), which was considered feasible and similar to that of humans. We adapted this simulator for the practice of proper suturing regarding second-degree perineal laceration. The second part, the hands-on clinical training, was divided into two steps: performing episiotomy on pig perineum and repairing second-degree perineal tears. Each participant was supervised by the trainers. Upon suturing completion, the medical students did a final assessment to confirm that the repair visually appeared to be accurate.

### Questionnaire survey

Medical students’ confidence levels after attending the workshop were measured with a questionnaire (5-point Likert scale, with 1 being extremely unconfident and 5 being extremely confident). From the medical students’ perspectives, the pig-perineum effectively simulated the vaginal canal,

anal canal, and anal sphincter, and the simulation training was helpful in instrument handling, knot typing and suturing of vaginal and perineal muscles. Moreover, the questionnaire also sought to evaluate their skills and satisfaction. The questionnaire was delivered immediately before and after the training session. The data obtained from the questionnaire were comprehensively reviewed after ethical approval.

### Statistical analysis

Descriptive data were presented as frequencies and percentages, means and SD or medians and interquartile ranges, as appropriate. The Wilcoxon signed rank test was used to compare the pre- and post-simulation training self-rated confidence levels of perineal repair skills. All data were recorded in an electronic database and subsequently analyzed, using the Statistical Package for the Social Sciences (SPSS) software, version 26.0 IBM Corp, released 2019 (IBM SPSS Statistics for Windows, Version 26.0 Armonk, NY: IBM Corp). A *p*-value of less than 0.05 was defined as statistically significant.

## Results

**Fig. 3:**
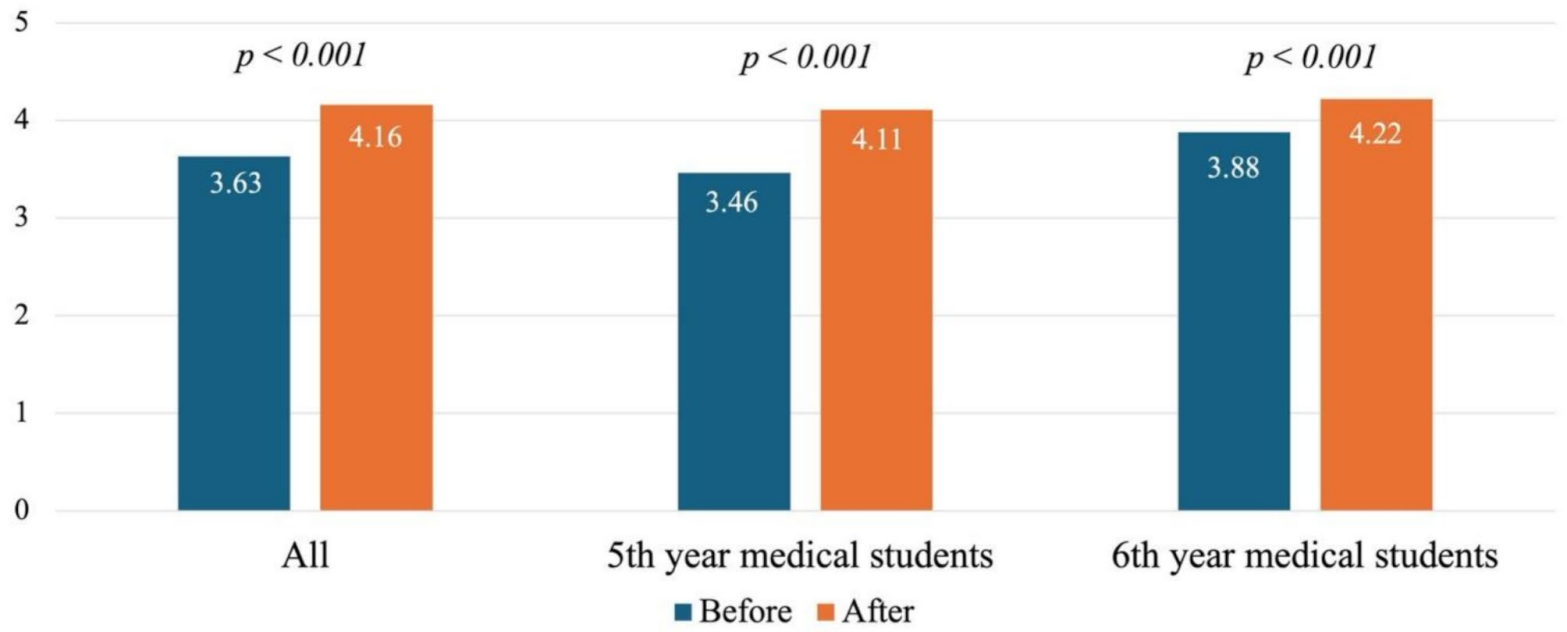
Confidence levels among medical students before and after attending the workshop (*N* = 792)

A total of 792 students participated in this pig-perineum simulation-based learning (SBL), including 475 (60%) 5th-year medical students and 317 (40%) 6th-year medical students. The survey included questions on confidence level, satisfaction, knowledge of perineal repair, and perspective on the closeness to reality of using pig perineum as a simulator for second-degree laceration repair. The mean confidence level among medical students before attending the workshop was 3.63 points. Notably, the lowest level of confidence was found among 5th year medical students (3.46 points). The assessment of the confidence level before and after training demonstrated a significant improvement of 10.6% in the mean score (*p*-value < 0.001), as shown in Fig. 3. Both pre- and post-training scores were higher for 6th-year medical students, but greater improvement was seen in 5th-year medical students. Figure 3 also displays the mean pre- and post-training confidence levels of the 5th- and 6th-year medical students.

**Fig. 4:**
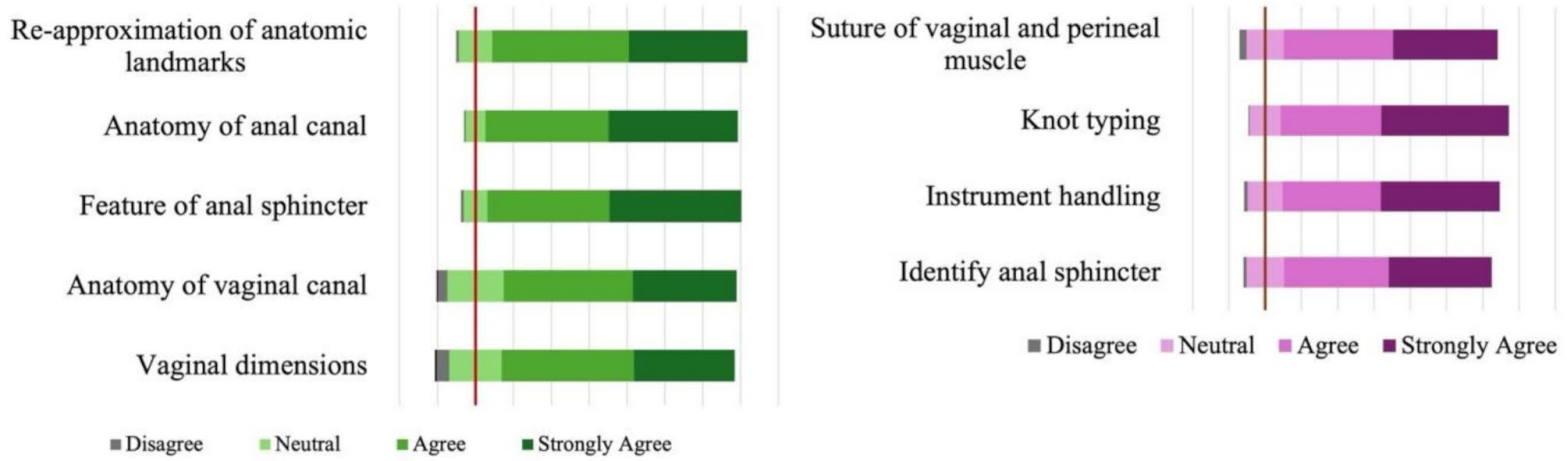
The closeness to reality of pig perineum as a simulator (*N* = 792)

Most medical students either strongly agreed or agreed that the pig-perineum simulator was effective in replicating the anatomical structures of the vaginal canal, perineal muscle and anal sphincter. Moreover, they would be able to utilize pig perineum for practising knot-tying and suturing perineal lacerations, which can enhance surgical skills and boost confidence after training. Figure 4 shows the details of their perspectives regarding the closeness to reality of pig perineum as a simulator.

Furthermore, the medical students gave high ratings for their performance skills, such as evaluation degree of perineal laceration and repairing 2nd-, 3rd-, and 4th-degree perineal lacerations, after attending the workshop. They were satisfied (mean satisfaction score = 4.22) with the training and asserted that the simulation improved their knowledge and surgical skills, which can be applied in their clinical practice. Table 1 shows the details of self-evaluation of the medical students regarding the hands-on pig-perineum SBL workshop for second-degree laceration suturing.

**Table 1:** Self-evaluation of the medical students regarding the hands-on pig-perineum simulation-based learning workshop for second-degree perineal laceration suturing

Evaluation items	Mean	SD	Median	Interquartile Range
Evaluation of perineal laceration	3.83	1.303	4	5
2nd degree perineal laceration repair	3.82	1.395	4	5
3rd degree perineal laceration repair	3.35	1.609	4	5
4th degree perineal laceration repair	3.32	1.624	4	5
Overall performance	3.65	1.513	4	5
Handling of surgical equipment	3.93	1.426	4	5
Application to real practice	4.16	1.31	5	5
Satisfaction score	4.22	1.317	5	5

## Discussion

Our study demonstrated that the medical students enrolled in hands-on pig-perineum SBL workshop showed an improvement of confidence level and satisfaction. Also, they had a positive attitude towards the pig-perineum simulator.

A recent survey reported that Thai medical graduates did not achieve the satisfactory confidence levels in performing normal vaginal delivery and episiotomy repair [5]. Additionally, tertiary medical schools are faced with a marked decrease in the number of vaginal deliveries all over the country. Thus, medical students currently have less experience than before, leading to even less confidence and competency in performing delivery and episiotomy repair. This emphasizes the necessity of providing more training opportunities for them.

SBL offers significant advantages, as it provides a comfortable atmosphere for learners to practice without the fear of making mistakes, thus giving them hands-on experience in a safe environment [14, 15]. Therefore, SBL helps medical students to rapidly improve their surgical skills [16]. As simulation becomes increasingly important in medical education, particularly for 3rd- and 4th-degree laceration repair, many training models have been used as simulators. Several studies have evaluated the effectiveness of teaching obstetric anal sphincter injury repair using practice models, and their results indicate improvement in resident skill sets after undergoing an educational workshop [17–19].

Among the types of simulation, those involving animals are the closest to real life because the tissues are similar to those of humans. Pig-perineum has been used for teaching OASI repair, and this study advocates the realistic and practical features of pig-perineum simulator in perineal repair training.

Pig-perineum SBL offers an attractive solution because it allows medical students to learn in a safe and controlled environment. For this reason, our obstetric and gynecological educational leadership team has made effort to achieve the use of pig-perineum simulators for perineal repair training. Students reported immediate increased comfort in assessing and repairing perineal lacerations, and they had high satisfaction levels and positive attitudes regarding this SBL. All the medical students stated that this simulation is valuable, and they would be able to utilize what they had learnt in clinical practice.

To the best of our knowledge, this is the first study that not only showed an improvement of medical students’ confidence level after a pig-perineum SBL for second-degree perineal lacerations but also inspired them to enhance their surgical skills in knot-tying and perineal laceration repair. Although there might be a significant gap between confidence and real proficiency, this study demonstrates the benefits of pig-perineum SBL in obstetric clerkship. Therefore, we strongly believe that a pig-perineum SBL program with realistic hands-on training should be incorporated in the obstetric curriculum of medical students.

The limitations of this study are as follows. Firstly, only the apprentices themselves evaluated the confidence levels and skills acquired in a single workshop, without assessment by validated tools. For future investigations, an evaluation, such as the Objective Structured Assessment of Technical Skills (OSATS), a theoretical test or the retention of knowledge, should be incorporated. Secondly, this study was based on self-reporting, which could lead to information bias. Lastly, the medical students were exposed to only one simulator intervention, without comparison with other training simulators. An alternative design should be allowed for comparison, to determine which simulator promotes efficiency and improves performance through skill acquisition better than the other.

The strengths of this study include: (1) A relatively large sample size of the participants, probably enhancing the reliability of the conclusion. (2) The high similarity of the simulator to human perineum in actual practice, in terms of tissue consistency and anatomical structures and their complexities. This is different from several previous studies, which used artificial models or tissues from other organs for the animal models (such as pig or beef tongue). Based on such similarity, the use of the model to practice the identification of anatomical structures, knot-tying, suturing and repair theoretically helps medical students to rapidly develop their skills in actual practice on patients, with less complications and shortened learning curve.

## Conclusion

Pig-perineum simulation-based learning improves confidence levels in undergraduate medical students. Theoretically, this approach is possibly helpful in enhancing competency during actual clinical practice.

## Electronic supplementary material

Below is the link to the electronic supplementary material.

Supplementary Material 1

## Data Availability

Data Availability Statement: The datasets analyzed during the current study are available from the corresponding author upon reasonable request.
