# Supplementary material for: Improving the confidence level and surgical skills of undergraduate medical students by using pig-perineum simulation-based learning
Source: BMC Med Educ. 2025 Feb 14;25:237. doi: 10.1186/s12909-025-06771-1 (PMC11827413; doi:10.1186/s12909-025-06771-1)
Supplement: Supplementary file 1 — Supplementary Material 1 [file 12909_2025_6771_MOESM1_ESM.docx]

Questionnaire for assessment of confidence level and surgical skill in undergraduate medical student by using pig-perineal simulation-based learning workshop

Department of Obstetrics and Gynecology, Chiang Mai University

**Part 1: Status** **of the participants**

- 5^th^ year medical student
  - Has experienced the labor ward
  - Has never experienced the labor ward
- 5^th^ year medical student
  - Has participated in an outside rotation
  - Has never participated in an outside rotation
  - Has attended this workshop
  - Has never attended this workshop

**Part  2: Reality of pig perineum** **compared to human perineum**

| **Items** | **Level of reality** | | | | |
| --- | --- | --- | --- | --- | --- |
|  | Strongly agree  5 | Agree  4 | Neither agree nor disagree  3 | Disagree  2 | Strongly disagree  1 |
| 1. Anatomy of pig perineum |  |  |  |  |  |
| - Vaginal dimensions |  |  |  |  |  |
| - Anatomy of vaginal canal |  |  |  |  |  |
| - Feature of anal sphincter |  |  |  |  |  |
| - Anatomy of anal canal |  |  |  |  |  |
| - Re-approximation of anatomic landmarks |  |  |  |  |  |
| 1. If you have experience with vaginal deliveries, do you think a pig's perineum is similar to a human's in real cases? |  |  |  |  |  |
| - Identifying anal sphincter |  |  |  |  |  |
| - Instrument handling |  |  |  |  |  |
| - Knot tying |  |  |  |  |  |
| - Suturing of vaginal and perineal muscle |  |  |  |  |  |

**Part 3: Confidence level and surgical skill to practice**

| **Items of assessment** | **Level of Confidence** | | | | |
| --- | --- | --- | --- | --- | --- |
|  | Strongly agree  5 | Agree  4 | Neither agree nor disagree  3 | Disagree  2 | Strongly disagree  1 |
| 1. Evaluation of perineal laceration |  |  |  |  |  |
| 1. Repairing 2^nd^ degree perineum laceration |  |  |  |  |  |
| 1. Overall performance |  |  |  |  |  |
| 1. 3^rd^ degree perineal laceration repair |  |  |  |  |  |
| 1. 4^th^ degree perineal laceration repair |  |  |  |  |  |
| 1. Handling of surgical instrument |  |  |  |  |  |
| 1. Application to actual practice |  |  |  |  |  |
| 1. Satisfaction score |  |  |  |  |  |
